# Co-Positivity for Anti-dsDNA, -Nucleosome and -Histone Antibodies in Lupus Nephritis Is Indicative of High Serum Levels and Severe Nephropathy

**DOI:** 10.1371/journal.pone.0140441

**Published:** 2015-10-14

**Authors:** Jinfeng Yang, Zhaozhen Xu, Manshu Sui, Jihua Han, Lijie Sun, Xiuzhi Jia, Haiyu Zhang, Changsong Han, Xiaoming Jin, Fei Gao, Yanhong Liu, Yang Li, Jianbin Cao, Hong Ling, Fengmin Zhang, Huan Ren

**Affiliations:** 1 Dept. of Immunology, Harbin Medical University, 150081 Harbin, China; 2 Immunity & Infection Key laboratory of Heilongjiang Province, 150081 Harbin, China; 3 Dept. of Clinical Laboratory Medicine, First Hospital Affiliated to Harbin Medical University, 150001 Harbin, China; 4 Dept. of Nephrology, First Hospital Affiliated to Harbin Medical University, 150001 Harbin, China; 5 Dept. of General Surgery, First Hospital Affiliated to Harbin Medical University, 150001 Harbin, China; 6 Dept. of Epidemiology and Biostatistics, Harbin Medical University, Harbin 150081, China; 7 Dept. of Pathology, Harbin Medical University, 150081 Harbin, China; 8 Dept. of Clinical Laboratory Medicine, Second Hospital Affiliated to Harbin Medical University, 150001 Harbin, China; 9 Dept. of Rheumatology, Second Hospital Affiliated to Harbin Medical University, 150001 Harbin, China; 10 Harbin center for disease control and prevention, 150081 Harbin, China; 11 Dept. of Microbiology, Harbin Medical University, 150081 Harbin, China; Instituto Nacional de Ciencias Medicas y Nutricion Salvador Zubiran, MEXICO

## Abstract

**Objective:**

To characterize the significance of correlated autoantibodies in systemic lupus erythematosus (SLE) and its complication lupus nephritis (LN) in a large cohort of patients.

**Methods:**

Clinical data were statistically analyzed in 1699 SLE patients with or without nephritis who were diagnosed and treated during 2002–2013 in the northeast region of China. Reactivity to a list of 16 autoantibodies was detected by the serum test Euroline ANA profile (IgG). Serum titers of the anti-nucleosome autoantibodies were measured by ELISA assays. Kidney biopsies were examined by pathologists. Immune complex deposition was identified by immunohistochemistry stain.

**Results:**

Simultaneous positivity of anti-dsDNA, -nucleosome and -histone antibodies (3-pos) was prevalent in SLE patients with LN compared to Non-renal SLE patients (41% vs 11%, *p*< 0.001). Significant correlations were found between any two of the above three anti-nucleosome antibodies in LN patients. In comparison to non-3-pos cohorts, 3-pos patients with LN had significantly higher serum levels of the three antibodies and more active disease; was associated with type IV disease; suffered from more severe renal damages; received more intensive treatment and had worse disease outcome. The serum levels of these three autoantibodies in 3-pos LN patients were significantly decreased when they underwent clinical recovery.

**Conclusions:**

Simultaneous reactivity to anti-dsDNA, -nucleosome and -histone antibodies by Euroline ANA profile (IgG) may indicate severe nephropathy in patients with SLE.

## Introduction

SLE is a typical autoimmune disease which diversely affects multiple end-organs, including heart, joints, liver and kidneys. Hyper activation of autoantibodies against cell nucleic antigens leads to the deposition of immune complex in end organs [1, 2]. LN is the most serious manifestation of SLE, occurring in 50–80% SLE patients. A substantial number of LN patients are refractory to conventional treatments, besides, renal relapse in LN patients is a risk factor for poor renal function [3, 4]. Therefore, early diagnosis of LN and more suitable treatments are highly warranted. Certain autoantibodies play an indicative role in SLE development. Serum test Euroline ANA Profile (IgG) is now routinely used to detect a series of autoantibodies against cell nuclei in serum of autoimmune diseases including SLE. Whereas a definite diagnosis on a specific autoimmune disease still relies on relevant laboratory and clinical examinations, at certain conditions, renal biopsies are also necessary.

Recent years, quantification on the serum levels and/or prevalence of one or more autoantibodies are demonstrated their significance in disease pathology. For example, Katsumata *et al* report that serum anti-C1q antibody level is positively associated with glomerular C1q deposition in LN [5]. Prevalence of anti-C1q, anti-dsDNA and anti-chromatin/nucleosome antibodies in Juvenile SLE (JSLE) patients is positively associated with LN and disease activity. Furthermore, these antibodies are sensitive and specific for diagnosis of JSLE [6]. Meanwhile, serum anti-α actin antibody seems to be a reliable biomarker for renal involvement in SLE patients, yet relevant antibody is not found in renal biopsy [7, 8]. We previously report that, in SLE patients, simultaneously positivity for anti-dsDNA, anti-nucleosome and anti-histone antibodies by Euroline ANA Profile (IgG) test is significantly relevant with LN onset and activity, and suggestive as a valuable indicator for renal involvement [9]. On the other side, many results demonstrate that anti-dsDNA antibody and other immune related components such as the levels of C3, C4 or anti-nucleosome antibody are negatively related with LN progression [10–14]. There are still no specific biomarkers that are publicly accepted for indicting SLE or LN pathology due to the variation of SLE population, region and measurements.

In this study, we collected and analyzed data of more than a decade (2002 to 2013) of SLE patients from Heilongjiang province, the northeast region of China, with a population of more than 38 millions. The region has a typical climate in the frigid-temperate zone and goes through three to four months’ frost-free period each year, which may relate to local morbidity of SLE and rheumatoid arthritis [15–17]. Our data confirmed that, simultaneous reactivity with anti-dsDNA, -nucleosome and -histone antibodies (3-pos) in patients with SLE were highly relevant to LN pathology. Three-pos LN patients showed significantly higher serum levels of these antibodies, suffered from more severe renal damage and needed more intensive treatments than non-3-pos LN patients, indicating 3-pos as an indicative biomarker for severe LN.

## Patients and Methods

### Ethical considerations

All participants provided written consent for study participation. This consent procedure and the study were reviewed and approved by the National Ethical Committee of the Public Health School of the Harbin Medical University, in compliance with the principles of the Helsinki Declaration II.

### Patient samples

All study participants attended SLE clinic at the 1st and 2nd Hospital Affiliated to Harbin Medical University from 2002 to 2013. All the patients with SLE met the American College of Rheumatology (ACR) classification criteria for SLE. 921 LN patients (854 females, 67 males, median age 35 years, range 9–80 years) and 778 patients without nephritis (724 females, 54 males, median age 34 years, range 10–80 years) were enrolled **(S1 Table)**. LN patients were classified using microscopic analysis of urinary sediments, 24 hour proteinuria, serum creatinine and complement C3 levels, in which 211 (23%) of LN patients (195 females, 16 males, age 35.09±24.38) were confirmed using renal biopsies as per the International Society of Nephrology (ISN) and WHO criteria for SLE nephritis. Peripheral blood serum (with 42 paired serum) was taken at diagnosis and remission (partial/complete) for measurement of serum autoantibodies, renal parameters including urinary sediment assessment, 24h urinary protein, Scr and blood urea nitrogen (BUN). Levels of C3 and C4 were determined by an automatic analyzer. **Disease activity was assessed by systemic lupus erythematosus disease activity index (SLEDAI).**

### Assays of anti-nuclear antibodies profile

ANA profile (mitochondrial-2, ribosomal-p, histone, nucleosome, dsDNA, PCNA, centromere, Jo-1, PM-Scl, Scl-70, SSB, Ro-52, SSA, sm, RNP) was detected by EUROLINE ANA profile (IgG) kit according to manufacturer’s instruction (EUROLINE, Lübeck, Germany). The results were read by EUROBlotMaster (Lübeck, Germany).

### Assays of anti-dsDNA, -nucleosome and -histone antibodies

Titres of the three antibodies were measured by ELISA using commercially available kits according to the manufacturer’s instruction (Demeditec, Bolin, Germany). The cutoff value was set at 20 U/ml, which was determined by the manufacturer.

### Evaluation of renal pathology

The renal biopsy specimens were examined by light microscopy and direct immunohistochemistry stain. Renal histopathology was classified according to the international society of nephrology/renal pathology society (ISN/RPS) 2003 revised criteria. Pathological parameters such as active indices (AI) and chronic indices (CI) were scored using a previously reported system involving semi-quantitative scoring of specific biopsy features.

Direct immunohistochemistry stain was used to detect IgG deposition in renal biopsy. Renal biopsies were stained with a horseradish peroxidase-labelled goat anti-human IgG antibody and a 3,3′-Diaminobenzidine (DAB) substrate to detect IgG expression. Section areas and positive cells were measured from digital images using image pro plus software (Media Cybernetics, Silver Springs, MD). The results were averaged and expressed as IOD/Aera tissue section.

### Renal treatment and outcome measurement

The patients were treated in accordance with clinical routine for LN [18]. The mainstay of induced therapy was prednisone (1 mg/kg per day) combined with cyclophosphamide (CTX, 0.75 g/m^2^ per month). Patients with severe necrotizing crescentic glomerulonephritis and diffuse pulmonary alveolar haemorrhage were additionally treated with methylprednisolone (MP, 7–15 mg/kg per day, 3 days) pulse therapy. For maintenance therapy, a low dose of prednisone (0.5 mg/kg per day) combined with immunosuppressive drugs such as cyclophosphamide (CTX, 0.75 g/m^2^ per every three month), azothioprine (1–2 mg/ kg per day), or mycophenolate mofetil (500–2000 mg per day) were administrated.

The remission of LN includes complete remission and partial remission. Complete remission was defined as urinary protein excretion < 0.3 g/day, normal urinary sediment (red blood cell < 3/ HP, white blood cell < 5/ HP), with normal serum albumin and normal renal function. Partial remission was defined as having one of the following items: decrease of Scr to < 130 μmol/L for patients with a baseline Scr level ≥ 130 μmol/L but ≤ 260 μmol/L; decrease of Scr by > 50% for patients with a baseline Scr level > 260 μmol/L; decrease of urinary protein excretion by > 50% and < 3.0 g/day, with a serum albumin level ≥ 30 g/L and stable renal function. A poor renal outcome was defined as a doubling in Scr values for a period of 6 months at least; with minimum value of the Scr at 176.8 μmol/L [19]. Renal relapse was refer to the doubling of the lowest Scr observed so far and/or developing either nephritic syndrome while the lowest proteinuria had been < 2.0 g/day repeatedly, or proteinuria > 1.5 g/day without other causes in a patient who didn’t have proteinuria.

### Statistical Analysis

Differences of quantitative parameters between groups were assessed using mann whitney U test. Differences of qualitative data were compared used chi-squared test or Fisher exact test. The odds ratio (OR) was calculated for assessing the risk of appearance of each variable. A lower limit of the 95% confidence interval (CI) that exceeded 1.0 was taken to indicate statistical significance in the case of positive association and an upper limit lower than 1.0 in the case of negative association. The Spearman Rank Correlation was used to analyze correlation and Bonferroni Correction was used to counteract the problem of multiple comparisons.

For the significant variables in the multivariate logistic regression analysis, we plotted receiver operating characteristic (ROC) curves to investigate the cutoff values of the significant variables to differentiate active LN from inactive LN. The area under the ROC curve (AUC) and 95% confidence intervals (CIs) were calculated. An AUC of 0.5 indicates chance performance, an AUC of 0.5–0.6 indicates poor predictive ability, an AUC of 0.6–0.7 indicates sufficient predictive ability, an AUC of 0.7–0.8 indicates good predictive ability, an AUC of 0.8–0.9 indicates very good predictive ability, and an AUC of 1.0 indicates excellent predictive ability [20].

All tests were used with two-sided options and significance level was set at a p value < 0.05. All statistical analyses were performed using the SPSS 20.0 statistics package (SPSS, Inc., Chicago, IL) and GraphPad Prism (GraphPad Software, Inc., SanDiego, CA, USA).

## Results

### Simultaneous positivity of anti-dsDNA, -nucleosome and -histone antibodies (3-pos) is prevalent in SLE patients with LN

Autoantibodies against specific antigens of cell nuclei in SLE patients were routinely tested by Euroline ANA Profile (IgG) kit in China. As we previously reported, in comparison to single, double, or null (3-neg) reactivity against anti-dsDNA, -nucleosome, or -histone antibodies, joint reactivity for these three antibodies (3-pos) were the most prevalent, occurring in 460 patients with lupus nephritis (LN) among 589 SLE patients from the single center [9]. In this study, we revised the profiles and applied analysis in a larger cohort, including 1699 SLE patients from the 1^st^ and 2^nd^ general hospitals affiliated to Harbin Medical University during 2002 to 2013. Of 921 SLE patients with LN, single positivity for anti-dsDNA, -nucleosome, or -histone antibodies presented in 583 (63.3%), 551 (59.8%), and 454 (49.3%) patients, respectively; and anti-mitochondrial-2 antibodies in 76 (8.3%) patients, all significantly higher than those in 778 SLE patients without nephritis. Meanwhile, anti-RNP and -centromere antibodies were positive in 76 (8.3%) and 24 (2.6%) LN patients, both significantly lower than those in non-LN patients (Table 1). Among those greatly elevated antibodies in LN patients, significant correlations by Pearson correlation analysis were identified between any two of anti-dsDNA, -nucleosome, or -histone antibodies, with the correlations between anti-dsDNA and -nucleosome antibodies (r = 0.709, *p*< 0.001); anti-histone and -dsDNA antibodies (r = 0.510, *p*< 0.001); and anti-histone and -nuclesome antibodies (r = 0.586, *p*< 0.001); In addition, anti-mitochondrial antibody showed significantly low or negative correlations with anti-dsDNA (r = 0.071, *p* = 0.001), -nucleosome (r = 0.076, *p* = 0.025) or -histone (r = 0.066, *p* = 0.052) antibodies. Anti-RNP and -centromere antibodies showed no correlation (r = 0.010, *p* = 0.763) with each other.

**Table 1:** Prevalence of autoantibodies in patients with SLE by Euroline ANA Profile (IgG).

Antibodies	LN (n = 921)	Non-renal SLE(n = 778)	OR (95% *CI*)	P value
ANA	897 (97.4%)	764 (98.2%)	0.685 (0.352–1.333)	0.263
**anti-mitochondrial-2**	76 (8.3%)	38 (4.9%)	1.751 (1.172–2.617)	**0.006**
anti-ribosomal-P	265 (28.8%)	209 (26.9%)	1.100(0.889–1.361)	0.382
**anti-histone**	454 (49.3%)	167 (21.5%)	3.557 (2.870–4.407)	**<0.001**
**anti-nucleosome**	551 (59.8%)	272 (35.0%)	2.770 (2.273–3.376)	**<0.001**
**anti-dsDNA**	583 (63.3%)	231 (47.9%)	4.084 (3.331–5.009)	**<0.001**
Anti-PCNA	21 (2.3%)	14 (1.8%)	1.273 (0.643–2.521)	0.487
**Anti- centromere**	24 (2.6%)	36 (4.6%)	0.551 (0.326–0.933)	**0.025**
Anti-Jo-1	5 (0.5%)	4 (0.5%)	1.056 (0.283–0.397)	1.000
Anti-PM-Scl	9 (1.0%)	3 (0.4%)	2.549 (0.688–9.450)	0.147
Anti-Scl-70	26 (2.8%)	30 (3.9%)	0.724 (0.425–1.236)	0.235
anti-SSB	139 (15.1%)	129 (16.6%)	0.894 (0.689–1.161)	0.402
anti-Ro-52	441 (47.9%)	343 (44.1%)	1.165 (0.962–1.411)	0.118
anti-SSA	484 (52.6%)	444 (57.1%)	0.833 (0.688–1.010)	0.062
anti-sm	207 (22.5%)	182 (23.4%)	0.949 (0.757–1.191)	0.654
**anti-RNP**	305 (33.1%)	309 (39.8%)	0.749 (0.614–0.913)	**0.004**

To further strengthen the joint reactivity of anti-dsDNA, -nucleosome or -histone antibodies in LN patients, odds ratio (OR) was calculated in different groups of patients within LN or non-LN patients. The OR value greater than one represents a risk factor, whereas less than one a protective factor [21]. Consistent with our previous results [9], the group of patients with joint reactivity of the three antibodies (3-pos) not only had the highest number of patients among different LN groups, but also significantly higher than that in non-LN patients, with 41.0% in 921 LN vs 11.2% in 778 non-LN patients, p< 0.001; in addition, 3-pos patients had the highest OR value of 5.529; 95% CI 4.269–7.162, in comparison to other groups with single, double or null reactivity of the three antibodies. These data indicated 3-pos as a high relevant risk factor to LN development. Further supporting this notion, null reactivity on the three antibodies (3-neg) was shown as the strongest protective factor to LN development, with the OR value of 0.313; 95% CI 0.258–0.383; and 3-neg patients in LN group (28.4%) significantly less than those in non-LN group (55.4%, p< 0.001, Table 2).

**Table 2:** Distribution of SLE patients with/without LN according to reactivity to anti-dsDNA, -nucleosome or -histone antibodies.

Anti-dsDNA	Anti-nucleosome	Anti-histone	LN (n = 921)	Non-renal SLE (n = 778)	OR (95%*CI*)	P value
Pos	Neg	Neg	62 (6.7%)	38 (4.9%)	1.406 (0.928–2.130)	0.107
Neg	Pos	Neg	22 (2.4%)	48 (6.8%)	0.372 (0.233–0.622)	**<0.001**
Neg	Neg	Pos	29 (2.1%)	26 (3.3%)	0.616 (0.338–1.122)	0.110
Pos	Pos	Neg	124 (13.5%)	95 (12.2%)	1.119 (0.840–1.489)	0.443
Pos	Neg	Pos	20 (2.2%)	12 (1.5%)	1.420 (0.690–2.924)	0.339
Neg	Pos	Pos	28 (3.0%)	42 (5.4%)	0.649 (0.337–0.895)	0.015
**Pos**	**Pos**	**Pos**	**378 (41.0%)**	**87 (11.2%)**	**5.529 (4.269–7.162)**	**<0.001**
Neg	Neg	Neg	258 (28.0%)	431 (55.4%)	0.313 (0.258–0.383)	**<0.001**

### Three-pos patients with LN have significantly higher serum levels of anti-dsDNA, -nucleosome, and -histone Abs, more active disease and is associated with the class IV nephropathy

**Fig 1:**
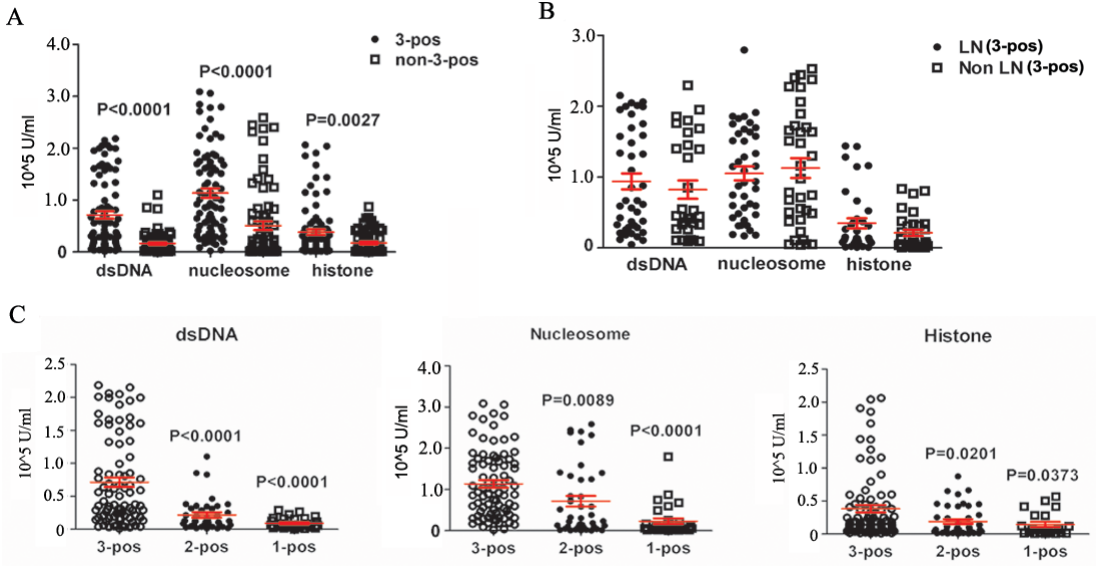
The serum Levels of anti-dsDNA, -nucleosome and -histone antibodies in SLE patients. **(A)** Serum levels of 3-pos and non 3-pos LN patients when they had active disease. **(B)** Serum levels of anti-dsDNA, -nucleosome, and -histone Abs in 3-pos SLE patients with/without LN. **(C)** Significantly higher titers of the three anti-nucleosome antibodies in 3-pos LN patients, compared to that in 2-pos or 1-pos cohorts. 3-pos: co-positivity of anti-dsDNA, -nucleosome, and -histone Abs; 2-pos: anti-dsDNA and -nucleosome, or anti-dsDNA and -histone, or anti -nucleosome and -histone; 1-pos: anti-dsDNA, or anti-nucleosome, or anti-histone. The antibody reactivity was determined by Euroline ANA profile (IgG).

To further evaluate the significance of 3-pos reactivity in LN pathology. Titers of anti-dsDNA, -nucleosome and -histone Abs in 3-pos and non-3-pos LN patients were measured, non-3-pos patients including patients whose serum showed single, double or null-reactivity against the three antibodies. The results showed that, respective serum levels of the three antibodies were significantly higher than that in non 3-pos LN group (*P*_*ds-DNA*_< 0.0001, *P*_*nuc*_< 0.0001, *P*_*his*_ = 0.0027) (Fig 1A). In addition, we randomly collected 3-pos serum samples of LN and non LN patients, and compared respective levels of the three antibodies. The results showed no significant difference (Fig 1B). Furthermore, within LN group, we also compared respective serum levels of the three antibodies between 3-pos and 2-pos (patients with joint reactivity against any two of the three antibodies), and 1-pos (only patients with single reactivity to one of the three antibodies) groups and confirmed significance in these comparisons (Fig 1C). These data implied that 3-pos reactivity may result in significantly higher disease activity regardless of renal involvement.

Consistent with significantly elevated serum levels of the three antibodies in 3-pos patients, the associated clinical and serological parameters including white blood cell count (WBC), and the complement fragments C3 and C4 (g/L) in 3-pos patients were significantly lower than that in non-3-pos patients, in addition to the decreased levels of hemoglobin (HB), C3 and C4, with both qualitative and quantitative tests (*p*< 0.0001 for all comparisons, Table 3).

**Table 3:** Baseline laboratory characteristics between 3-pos and non 3-pos patients with LN.

Index	3-pos (378)	non 3-pos (543)	*P* value
Age (years)	33 (22.75–44.00)	35 (25.00–44.00)	0.336
Female	349 (92.3%)	496 (91.3%)	0.594
**Urinary protein (g/24h)**	4.94 (1.41–7.04)	2.29 (0.88–5.84)	**<0.001**
**Urinary RBC (n/HP)**	9.95 (2.35–34.62)	4.03 (1.61–15.36)	**<0.001**
**Urinary WBC (n/HP)**	50.90 (23.00–151.00)	25.30 (12.30–74.50)	**<0.001**
**SCr (μmol/L)**	81.00 (63.95–120.15)	65.50 (54.90–87.10)	**<0.001**
**BUN (mmol/L)**	7.52 (5.15–12.63)	5.92 (4.37–8.74)	**<0.001**
WBC (×10^9^/L)	4.43 (2.96–7.00)	5.80 (3.73–8.61)	**<0.001**
PLT (×10^9^/L)	165.00 (110.00–229.00)	177.00 (108.00–239.00)	0.436
HB (g/L)	62.00 (3.38–101.00)	52.50 (3.68–108.00)	**0.017**
**C3 (g/L)**	0.39 (0.27–0.55)	0.54 (0.35–0.83)	**<0.001**
**C4 (g/L)**	0.06 (0.03–0.10)	0.09 (0.05–0.17)	**<0.001**
Decreased HB (≦110g/L)	285/378 (75.4%)	290/543 (53.4%)	**<0.001**
Decreased C3 (≦0.88g/L)	320/333 (96.1%)	390/495 (78.8%)	**<0.001**
Decreased C4 (≦0.10g/L)	254/333 (76.3%)	281/495 (56.8%)	**<0.001**
ANA (+)	378/378 (100%)	519/543 (95.6%)	**<0.001**
Anti-SSA (+)	209/378 (55.3%)	258/543 (47.5%)	**0.020**
Anti-SSB (+)	73/378 (19.3%)	86/543 (15.8%)	0.170
Anti-Sm (+)	89/378 (23.5%)	117/543 (21.5%)	0.474
Anti-RNP (+)	119/378 (31.5%)	190/543 (35.0%)	0.267
Anti-ribosomal-P (+)	119/378 (31.5%)	146/543 (26.9%)	0.130

**Values were shown as median (IQR) or number (%). IQR: inter-quartile range**.

**Fig 2:**
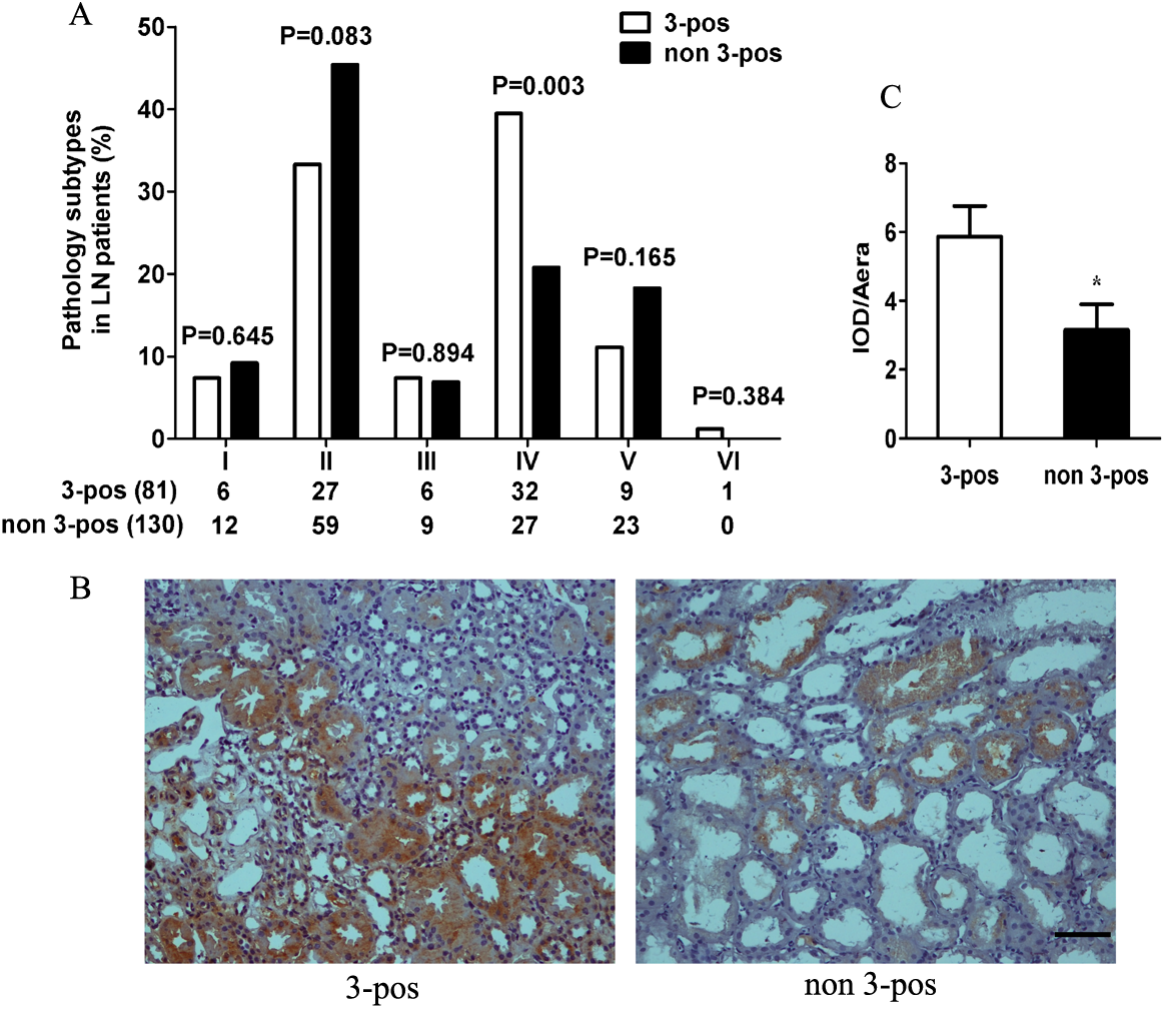
Correlation of 3-pos with LN subtype and kidney immune complex deposition in type IV LN. **(A)** Correlations of 3-pos with LN subtype. **(B)** Deposition of immune complex in type IV LN in 3-pos or non-3-pos patients by immunohistochenistry. **(C)** Quantification of the postive staining in **(B)**. IgG was labeled by HRP and represented for immune complex deposition. Four fields per biopsy sample were randomly counted and results were expressed as IOD/Area (Mean±SEM). Scale bar = 50 μm.

Moreover, on examining the pathological diagnosis on 211 LN patients (clinical and lab characters shown in S2 Table) whose renal biopsies were taken, we found that, 3-pos patients seriously tended to suffer from type IV nephritis, as compared to non-3-pos patients (*p* = 0.003, Fig 2A). IgG immune complex deposition on renal biopsy was further investigated the by immunohistochemistry stain. We randomly counted 4 fields per biopsy sample (total 211) and results were expressed as IOD/Area (Mean±SEM). There was significantly greater renal deposition of IgG immune complexes in 3-pos patients’ biopsies (5.87±0.89) than that of non-3-pos patients’ (3.16±0.74), both diagnosed with type IV nephritis (Fig 2B & 2C, *p*< 0.05). Collectively, these data indicated significantly more intensive immune response and higher disease activity occurred in 3-pos LN patients.

### Three-pos patients with LN are suffered from more severe renal damage and worse disease outcome than non-3-pos patients

**Fig 3:**
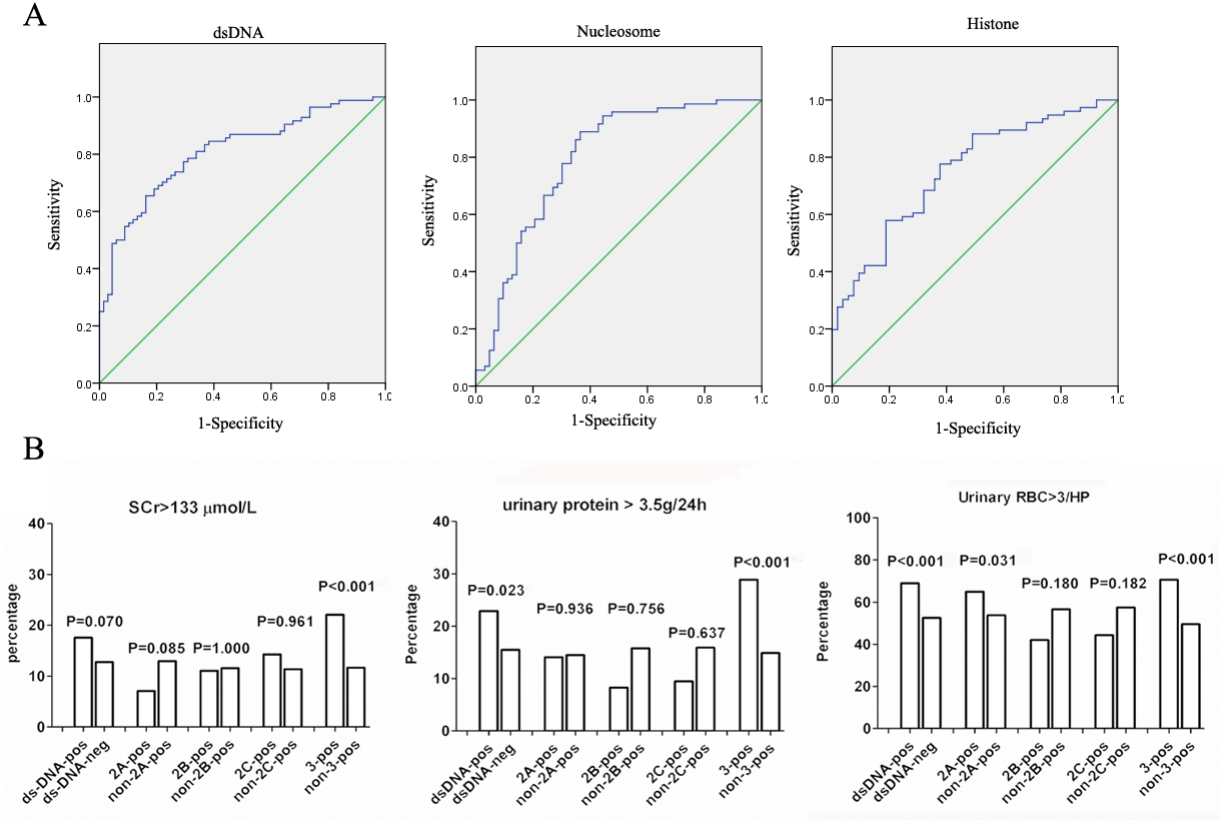
Three-pos by Euroline ANA profile (IgG) was a marker for severe nephropathy in LN patients. **(A)** Power of anti-dsDNA, -nucleosome and–histone Abs to differentiate active LN from inactive LN accessed by ROC curve. **(B)** Three-pos was the best marker for severe nephropathy. The relevant laboratory parameters on nephropathy includes increased Scr (≥ 133μmol/L), urinary protein (≥ 3.5g/24h) and urinary RBC (≥ 3/HP). 2A, 2B and 2C respectively indicated varied 2-pos by Euroline ANA profile (IgG) in LN patients; 2A: anti-dsDNA and -nucleosome; 2B: anti-dsDNA and -histone; 2C: anti-nucleosome and -histone.

Additional comparisons on the renal function-associated clinical parameters confirmed an indicative role of 3-pos in the cohort of patients with more severe nephritis. For a qualitative evaluation, the medians of 24 h urine protein excretion, Scr, BUN, urinary RBC and urinary WBC were all significantly higher in 3-pos group than those in non 3-pos patients (*p*< 0.0001 for all comparisons) (Table 3). We next divided LN patients into active LN (SLEDAI>0, 24 hour urinary protein > 0.5g, elevated SCr and BUN) and inactive LN (SLEDAI = 0, with no urinary protein, normal SCr and BUN). The predictive ability of anti-dsDNA, -nucleosome and -histone Abs to differentiate active LN from inactive LN was measured by ROC curve. ROC analysis revealed AUCs for the anti-dsDNA, -nucleosome and–histone Abs of 0.809 (95% CI = 0.741–0.877, *p*< 0.001), 0.792 (95% CI = 0.731–0.871, *p*< 0.001) and 0.750 (95% CI = 0.666–0.834, *p*< 0.001), respectively (Fig 3A). The optimal cutoff value for anti-dsDNA Ab for predicting active LN was 16.89 (1000 U/ml) (sensitivity = 70.0%, specificity = 78.0%). The optimal cutoff value for anti-nucleosome Ab for predicting active LN was 49.62 (1000 U/ml) (sensitivity = 70.8%, specificity = 71.4%). The optimal cutoff value for anti-histone Ab for predicting active LN was 16.60 (1000 U/ml) (sensitivity = 68.4%, specificity = 68.0%). Moreover, to emphasize the best indicative role of 3-pos for more severe nephritis, we increased the levels of certain key parameters that may define further disturbed renal activity or damages, including Scr (≥ 133 μmol/L), urinary protein (≥ 3.5 g/24 h) and urinary RBC (≥ 3/HP), and made comparisons between 3-pos and non-3-pos, 2-pos and non-2-pos, or 1-pos and non-1-pos patients. The data indicated that, significant differences were only obtained in the comparison between 3-pos and non-3-pos patients (*p*< 0.001 for all), but not in other two comparisons (Fig 3B).

Moreover, to show if 3-pos were linked with certain pathological features in renal biopsies, we analyzed the correlations of characteristics of activity indices (AI) and chronicity indices (CI) with 3-pos LN patients. The results showed that, 3-pos reactivity was significantly correlated with AI but not CI (for AI, r = 0.1954, *p* = 0.0243; for CI, r = 0.09764, *p* = 0.2717). In more details, 3-pos reactivity in LN patients was greatly associated with a few typical AI features including *endocapillary hypercellularity* (r = 0.1749, *p* = 0.0432), *glomerular leukocyte infiltration* (r = 0.1858, *p* = 0.0316), and *wire-loop formation* (r = 0.1892, *p* = 0.0285) (S3 Table). These data kept close consistency with those indicating that, 3-pos LN patients had a greatly higher tendency to develop class IV nephritis than non-3-pos cohorts (Fig 2A).

Renal damages in LN patients were frequently linked with disease recurrence and outcome. Consistently, the recurrent rate of 3-pos LN patients was significantly higher than that of non 3-pos patients (62.4% vs 26.5%, *p*< 0.001). Moreover, the remission rate in 3-pos LN patients was greatly lower than non 3-pos patients (62.2% vs 83.8%, *p*< 0.001); in addition to the number of patients with poor outcome (71.1% vs 4.4%, *p*< 0.001; Table 4). Taken together, these data all pointed out 3-pos reactivity in LN patients as an indicator of a significantly more severe nephropathy.

**Table 4:** Disease outcome in 3-pos and non 3-pos patients with LN.

Clinical treatment and outcomes	3-pos (378)	Non 3-pos (543)	P value
**Relapse rate**	236 (62.4%)	144 (26.5%)	**<0.001**
**Remission rate**	256 (62.2%)	451 (83.8%)	**<0.001**
Mortality rate	17 (4.6%)	26 (4.8%)	0.771
ESRD rate	26 (6.9%)	34 (6.2%)	0.503
**Patients with poor outcome**	38 (10.1%)	24 (4.4%)	**<0.001**

### After intensive treatments, serum titres of anti-dsDNA, -nucleosome, and -histone Abs in 3-pos LN patients are significantly dropped when they underwent clinical recovery

Steroids and immunosuppressant were the most frequently used drugs in treating autoimmune disease. In LN patients, these drugs were often administrated. Because we well demonstrated in this study, 3-pos reactivity of the three antibodies was an indicator of a high disease activity and more severe renal dysfunction in LN patients, we next analyzed differences of the treatments between 3-pos and non-3-pos patients after their initial diagnosis with nephritis. The data showed that, significantly more intensive treatments were administrated to 3-pos patients than non-3-pos regarding the frequency of drug administration and dosages. Of the 378 LN patients with 3-pos, 170 (44.9%) used prednisone, 138 (36.7%) used methylprednisone sodium succinate, 62 (16.5%) used methyprednisolone pulse therapy (≥ 500/mg), and 46 (12.2%) used cyclophosphamide. These drugs were more frequently used in 3-pos than non 3-pos LN patients. Furthermore, greatly higher administration dosages of certain drugs were also applied in 3-pos group (Table 5).

**Table 5:** Clinical treatments between 3-pos and non 3-pos patients with LN.

Drug selection and dosage	3-pos (378)	Non 3-pos (543)	P value
**Drugs**			
**Prednisone**	170 (44.9%)	155 (28.6%)	**<0.001**
**Methylrednisone sodium succinate**	138 (36.7%)	134 (24.8%)	**0.005**
Hydroxychloroquine sulfate	46 (12.2%)	63 (11.6%)	0.688
Methylprednisone	287 (76.1%)	393 (72.4%)	0.667
Leflunomide	26 (6.9%)	21 (3.8%)	0.063
**Methylprednisone pulse therapy (>500/mg)**	62 (16.5%)	42 (7.8%)	**0.003**
Mycophenolate	98 (26.1%)	129 (23.8%)	0.462
**Cyclophosphamide**	46 (12.2%)	33 (6.1%)	**0.007**
Dexamethasone	14 (3.9%)	10 (1.9%)	0.146
**Dosage**			
Prednisone/mg	140 (70.00–365.60)/ mg	120 (70.00–180.00)/ mg	0.103
**Methylrednisone sodium succinate/mg**	90 (60.00–130.00)/ mg	60 (40.00–85.00)/ mg	**<0.001**
**Methylprednisone pulse therapy (>500/mg)**	25 (20.00–40.00)/ mg	20 (10.00–30.00)/ mg	**0.004**
Cyclophosphamide/mg	445 (223.29–666.71)/ mg	457.14 (229.39–684.89)/ mg	0.715

**Fig 4:**
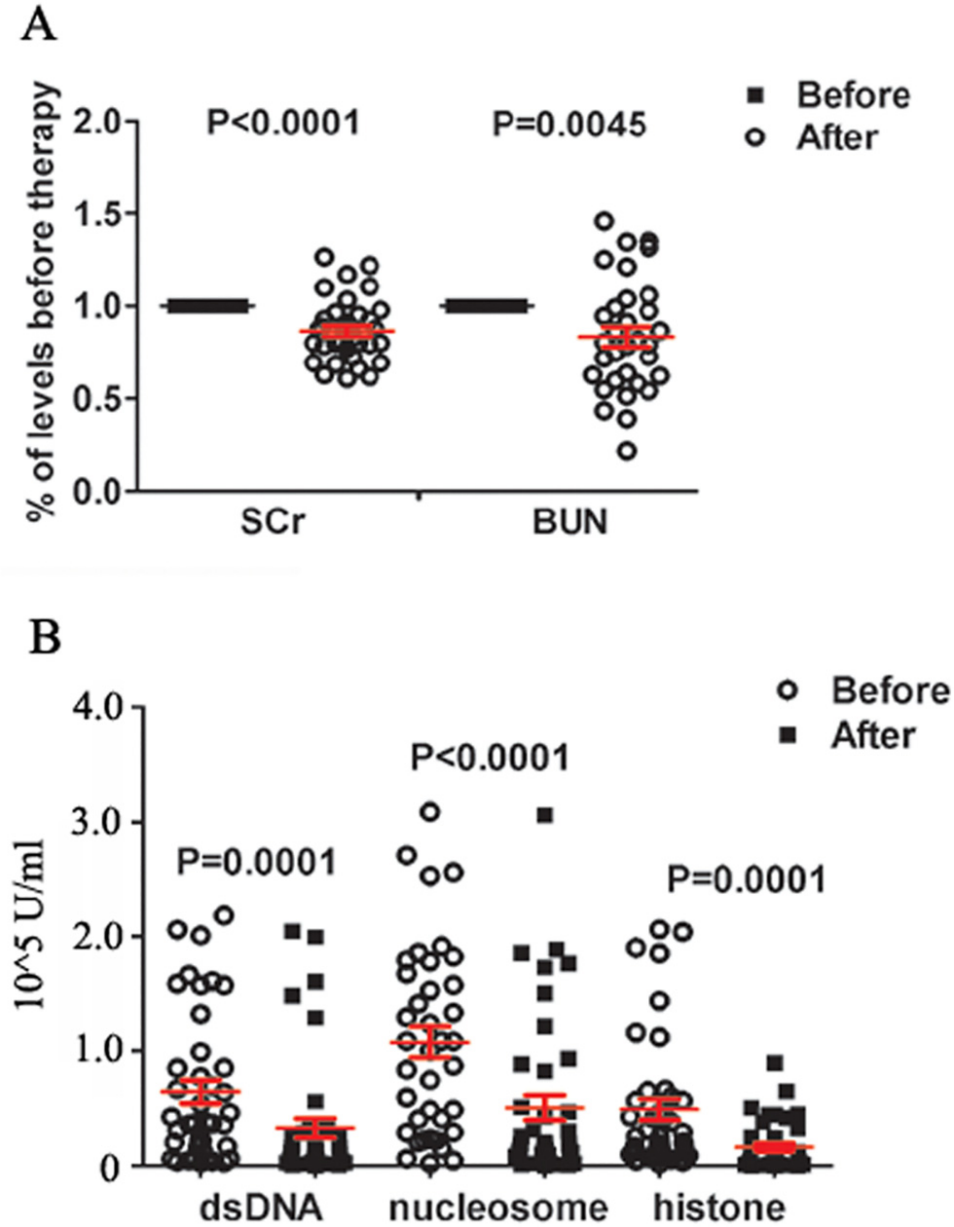
Serum titres of anti-dsDNA, -nucleosome, and -histone Abs in 3-pos LN patients are significantly dropped when they underwent clinical recovery. **(A)** Three-pos LN patients of clinical recovery had significantly decreased levels of SCr and BUN. **(B)** Serum levels of anti-dsDNA, -nucleosome and -histone antibodies in 3-pos LN patients before and after clinical treatment.

To further evaluate correlation of the antibody titres in 3-pos LN patients when they underwent clinical recovery, paired serum of 42 patients were randomly collected at the time when they had their initial diagnosis and got partial or complete remission (Judged according to the criteria in *patients and methods* part) after drug treatments in one disease duration. Clinical recovery of these patients was judged by evaluating relevant laboratory examinations (i.e. values of SCr and BUN at 24 h etc.) and the patients’ clinical symptoms. After therapy both SCr and BUN levels of these patients were dropped back to normal or showed a significant decrease (Fig 4A), and their clinical symptoms were greatly eased judged by doctors or discharged from the hospital. Consistently, titres of anti-dsDNA, -nucleosome, -histone antibody were all significantly decreased in these 3-pos- patients when they underwent clinical recovery and remission of the disease (*p*< 0.001 for all, Fig 4B). Collectively, these data further strengthened an indicative role of 3-pos reactivity in LN severity.

## Discussion

In this study, we retrospectively analyzed characterization of a series of clinical data with a large cohort of 1699 SLE patients in the northeast region of China during 2002 to 2013; among these patients, 921 were diagnosed with LN. Consistent with our previous report, we concluded that, simultaneous reactivity with anti-dsDNA, -nucleosome and -histone antibodies (3-pos) in patients with SLE may be used as a marker for high disease activity, especially, a more severe form of LN, in comparison to non-3-pos cohorts.

LN had been proposed as the prototype of an immune complex disease [22], in which autoantibodies directed against nuclear components were the most characteristic. These include autoantibodies against dsDNA, nucleosomes and histones [23]. For a list of 16 autoantibodies that were detected by Euroline ANA Profiles in our patients when they underwent active SLE, specific autoantibodies against these nuclear components were significantly correlated; more importantly, simultaneous reactivity to these antibodies was prevalent and positively correlated to LN development, with the odds ratio of 5.529. Recently, a series of relevant studies by Berden et al. showed that nucleosomes were a major driving force in the formation of antinuclear antibodies, among which, anti-dsDNA and -nucleosome antibodies were highly nephritogenic [24, 25]. Consequently, anti-nucleosome antibodies occurred early in life, before appearance of anti-dsDNA and anti-histone antibodies in lupus mice [26, 27]. Based on data with animal models and human samples of LN, they demonstrated that, only anti-nucleosome antibody complexes, especially with anti-DNA antibody complexes, rather than single specificity antibodies, can bind glomerular basement membrane (GBM) *in vivo* and induce proteinuria [26–28]. Furthermore, while eluted IgG of the glomerular-deposited antibodies from glomeruli of MRL/*lpr* lupus mice contained anti-nucleosome, -dsDNA and -histone antibodies; the amount of these antibodies was positively associated with the onset of proteinuria [29]. Thus, the presence and nephritogenicity of nucleosome-containing immune complexes were verified in kidney biopsies of murine and human LN [30–32]. Collectively, these and other relevant data showed the link between a sequential & quantitative production of these anti-nucleosome antibodies and LN pathology, indicating inherent correlations among the three autoantibodies during the course of disease [13, 33].

Moreover, a number of clinical studies indicated that, the level of individual autoantibodies, including anti-nucleosome and anti-dsDNA antibodies were strongly associated with activity of LN [34, 35], especially, anti-nucleosome antibodies were previously described to be a marker of active LN. In this study, while we found 3-pos patients suffered from more severe nephropathy than non-3-pos patients, the level of these three antibodies were significantly decreased when these patients underwent clinical recovery after intensive treatment. Kept consistent with our previous findings, our data illustrated the correlation and dynamics of these anti-nucleosome antibodies in LN pathology in a large cohort of patients. From these observations, however, we may not rule out that these 3-pos patients still kept 3-pos in their remission stage, because Euroline ANA profiles was only tested for initial diagnosis but not when patients were discharged or re-admitted to the same hospital in China. Thus, our data suggested that, 3-pos reactivity to these three antibodies by Euroline ANA profiles (IgG) may recognize a threshold of a high disease activity, most likely resulted from a soaring immune response against the release of nuclear components, and indicate a worse disease outcome. Nonetheless, it is trustworthy to conduct further prospective studies, following these antibodies over time from serial bleeds, to better understand the pattern of key autoantibodies during disease, and thus, define the best treatment strategy.

It had been recognized that patients with SLE could be divided into more homogeneous subsets of pathogenic, therapeutic, and prognostic status [36]. To our best knowledge, we were the first to analyze the role of correlated rather than individual autoantibodies in SLE pathology with large clinical data. These data were collected over a decade in an area of the frigid-temperate zone, which, as an environmental factor, may contribute to regional morbidity of the disease and complications. Furthermore, these clinical data were from two big hospitals that affiliated to Harbin Medical University, it was thus assumed that the diagnosis and treatment criteria kept standard, and thus, the conclusion was more consistent. Nonetheless, our results should be confirmed with other sets of data from other area of China with different location and climate, or worldwide. In addition, the list of autoantibodies by Euroline ANA profile (IgG) should include other key antibodies, i.e. anti-C1q etc. More prospective studies are needed and currently ongoing in our center. In conclusion, 3-pos reactivity with the anti-nucleosome autoantibodies by non-invasive Euroline ANA profile (IgG) test is an indicator of severe nephropathy in SLE.

## Supporting Information

S1 Table Systemic lupus erythematosus patient demographics. (DOC)

S2 Table Baseline laboratory characteristics of LN patients with/without renal biopsy. (DOC)

S3 Table Correlations between 3-pos and the relevant histopathological parameters. (DOC)

S1 Text Basic information and ANA profile of patients. (XLS)
