# Supplementary material for: Co-Positivity for Anti-dsDNA, -Nucleosome and -Histone Antibodies in Lupus Nephritis Is Indicative of High Serum Levels and Severe Nephropathy
Source: PLoS One. 2015 Oct 14;10(10):e0140441. doi: 10.1371/journal.pone.0140441 (PMC4605492; doi:10.1371/journal.pone.0140441)
Supplement: S1 Table — (DOC) [file pone.0140441.s001.doc]

**S1 Table. Systemic lupus erythematosus patient demographics**

| Demographics | SLE |
| --- | --- |
| Age (at diagnosis) (mean±SD) (range) | 22.6±16.3 yr (9-48) |
| Age (at enrollment) (mean±SD) (range) | 34.9±13.5 yr (10-80) |
| Gender (female/male) (%female) | 1578/121 (92.8%) |
| Disease duration (mean±SD) (range) | 17.4±8.7 yr (onset-32) |
| SLEDAI 2K score (range) (%of positive) | 0-23 (1254/73.8%) |

SLEDAI 2K positivity based on active disease defined at a score of 4.
