# Supplementary material for: Co-Positivity for Anti-dsDNA, -Nucleosome and -Histone Antibodies in Lupus Nephritis Is Indicative of High Serum Levels and Severe Nephropathy
Source: PLoS One. 2015 Oct 14;10(10):e0140441. doi: 10.1371/journal.pone.0140441 (PMC4605492; doi:10.1371/journal.pone.0140441)
Supplement: S2 Table — (DOC) [file pone.0140441.s002.doc]

**S2 Table. Baseline laboratory characteristics of LN patients with/without renal biopsy**

| Index | Renal biopsy (211) | LN (921) | *P* value |
| --- | --- | --- | --- |
| Age (years) | 32 (21.75-43.00) | 34 (22.75-44.00) | 0.236 |
| Female | 195 (92.4%) | 845 (91.7%) | 0.482 |
| **Urinary protein (g/24h)** | 3.71 (1.66-6.04) | 3.62 (0.88-7.04) | 0.354 |
| **Urinary RBC (n/HP)** | 6.85 (0–879.13) | 6.99 (1.61–846.70) | 0.409 |
| **Urinary WBC (n/HP)** | 35.40 (20.00-67.00) | 38.10 (12.30-81.00) | 0.231 |
| **SCr (μmol/L)** | 75.70 (57.93-80.76) | 73.25 (54.90-120.15) | 0.594 |
| **BUN (mmol/L)** | 6.46 (5.36-13.21) | 6.72 (4.37-12.63) | 0.351 |
| WBC (×10^9^/L) | 4.86 (1.48-8.15) | 5.12 (2.96-8.61) | 0.239 |
| PLT (×10^9^/L) | 162.00 (105.00-240.00) | 171.00 (108.00-239.00) | 0.392 |
| HB (g/L) | 54.00 (4.19-105.00) | 57.25 (3.38-108.00) | 0.405 |
| **C3 (g/L)** | 0.40 (0.13-0.45) | 0.47 (0.27-0.83) | 0.658 |
| **C4 (g/L)** | 0.05 (0.02-0.08) | 0.07 (0.03-0.17) | 0.357 |
| Decreased HB (≦110g/L) | 144/211 (68.1%) | 575/921 (62.4%) | 0.192 |
| Decreased C3 (≦0.88g/L) | 164/192 (85.4%) | 710/828 (85.7%) | 0.552 |
| Decreased C4 (≦0.10g/L) | 125/192 (65.1%) | 535/828 (64.6%) | 0.402 |

WBC, white blood cell; RBC, red blood cell; SCr, serum creatinine; BUN, blood urea nitrogen; PLT, platelet; HB, haematoglobin.
