# Supplementary material for: Co-Positivity for Anti-dsDNA, -Nucleosome and -Histone Antibodies in Lupus Nephritis Is Indicative of High Serum Levels and Severe Nephropathy
Source: PLoS One. 2015 Oct 14;10(10):e0140441. doi: 10.1371/journal.pone.0140441 (PMC4605492; doi:10.1371/journal.pone.0140441)
Supplement: S3 Table — (DOC) [file pone.0140441.s003.doc]

**S3** Table. Correlations between 3-pos and the relevant histopathological parameters

| Histopathologic parameters | Correlation coefficient (*r*) | *P* |
| --- | --- | --- |
| AI | 0.1954 | **0.0243** |
| Endocapillary hypercellularity | 0.1749 | **0.0432** |
| Glomerular leukocyte infiltration | 0.1858 | **0.0316** |
| Cellular crescents | 0.1092 | 0.2109 |
| Karyorrhexis/fibronoid necrosis | 0.1174 | 0.1783 |
| Formation of wire-loop | 0.1892 | **0.0285** |
| Infiltraion of interstitial inflammatory cells | -0.0283 | 0.7451 |
| CI | 0.0967 | 0.2717 |
| Glomerular sclerosis | 0.0341 | 0.6983 |
| Fibrous crescents | 0.0352 | 0.6890 |
| Atrophy of renal tubule | 0.0463 | 0.5983 |
| Interstitial fibrosis | 0.1769 | **0.0425** |

AI, activity index; CI, chronicity index
